# Genotype, Mortality, Morbidity, and Outcomes of 3β-Hydroxysteroid Dehydrogenase Deficiency in Algeria

**DOI:** 10.3389/fendo.2022.867073

**Published:** 2022-06-10

**Authors:** Asmahane Ladjouze, Malcolm Donaldson, Ingrid Plotton, Nacima Djenane, Kahina Mohammedi, Véronique Tardy-Guidollet, Delphine Mallet, Kamélia Boulesnane, Zair Bouzerar, Yves Morel, Florence Roucher-Boulez

**Affiliations:** ^1^Department of Paediatrics, Centre Hospitalo-Universitaire Bab El Oued, Algiers, Algeria; ^2^Section of Child Health, School of Medicine, Queen Elizabeth University Hospital, Glasgow, United Kingdom; ^3^Molecular Endocrinology and Rare Diseases, Hospices Civils de Lyon, Lyon University Hospital, Bron-Lyon, France; ^4^Department of Pathological Anatomy, Centre Hospitalo-Universitaire Bab El Oued, Algiers, Algeria

**Keywords:** 3-β hydroxysteroid dehydrogenase deficiency, 3βHSD2, HSD3B2, congenital adrenal hyperplasia, newborn screening, adrenal rest tumors, polycystic ovary syndrome

## Abstract

**Background:**

3β-hydroxysteroid dehydrogenase 2 (3βHSD2) deficiency is a rare form of congenital adrenal hyperplasia (CAH), with fewer than 200 cases reported in the world literature and few data on outcomes.

**Patients and Methods:**

We report a mixed longitudinal and cross-sectional study from a single Algerian center between 2007 and 2021. Virilization and under-masculinization were assessed using Prader staging and the external masculinization score (EMS), pubertal development staged according to the system of Tanner. Adrenal steroids were measured using mass spectrophotometry (LC-MS/MS). A genetic analysis of *HSD3B2* was performed using Sanger sequencing.

**Results:**

A 3βHSD2 defect was confirmed in 6 males and 8 females from 10 families (8 consanguineous), with p.Pro222Gln mutation in all but two siblings with a novel deletion: c.453_464del or p.(Thr152_Pro155del). Probable 3βHSD2 deficiency was diagnosed retrospectively in a further 6 siblings who died, and in two patients from two other centers. In the genetically confirmed patients, the median (range) age at presentation was 20 (0–390) days, with salt-wasting (n = 14) and genital anomaly (n = 10). The Prader stage for female patients was 2 (1–2) with no posterior fusion of the labia. The EMS for males was 6 (3–9). Median (range) values at diagnosis for 17-hydroxyprogesterone (17-OHP), dehydroepiandrosterone sulfate (DHEA-S), and 17-hydroxypregnenolone (17OHPreg) were elevated: 73.7 (0.37–164.3) nmol/L; 501.2(9.4–5441.3) nmol/L, and 139.7 (10.9–1500) nmol/l (NB >90 nmol/L diagnostic of 3βHSD2 defect). Premature pubarche was observed in four patients (3F:1M). Six patients (5F:1M) entered puberty spontaneously, aged 11 (5–13) years in 5 girls and 11.5 years in one boy. Testicular adrenal rest tumors were found in three boys. Four girls reached menarche at 14.3 (11–14.5) years, with three developing adrenal masses (surgically excised in two) and polycystic ovary syndrome (PCOS), with radiological evidence of ovarian adrenal rest tumor in one. The median IQ was 90 (43–105), >100 in only two patients and <70 in three.

**Conclusions:**

The prevalence of 3βHSD2 deficiency in Algeria appears high, with p.Pro222Gln being the most frequent mutation. Mortality is also high, with significant morbidity from adrenal tumors and PCOS in adolescence and an increased risk of learning disability. The finding of adrenal tumors in older patients with 3βHSD2 indicates under-replacement, requiring effective hydrocortisone and fludrocortisone treatment rather than surgical removal.

## Introduction

3β-hydroxysteroid dehydrogenase type 2 (3βHSD2) deficiency is a rare cause of congenital adrenal hyperplasia (CAH) with an estimated birth prevalence of less than 1/1,000,000 (1) and with fewer than 200 families reported in the world literature (2). The condition is transmitted in an autosomal recessive pattern and results from mutations in the *HSD3B2* gene, which encodes the type II 3βHSD isoenzyme (3). With a severe *HSD3B2* gene defect, biosynthesis of all steroids—mineralocorticoid, glucocorticoid, and sex hormones—is impaired, resulting in varying degrees of salt-wasting (SW), and under-masculinisation in 46, XY individuals. The phenotype of 3βHSD deficiency is linked to the type of *HSD3B2* mutation and to the residual activity of the 3βHSD enzyme. Thus, as in 21-hydroxylase deficiency (21OHD), classical salt-wasting forms have been described in 3βHSD deficiency as well as classical non-salt-wasting forms presenting with isolated under-masculinisation in 46,XY individuals (4). In affected women, virilization is usually absent or limited to clitoral enlargement. No *HSD3B2* mutation has been found in presumed non-classical forms with milder hyperandrogenism (5). While testicular adrenal rest tumor is well-recognized in 21OHD and has also been described in the ovaries of female patients with this variety of CAH (6–9), there have been only rare reports of adrenal rests in 46, XY patients with 3βHSD2 deficiency. Moreover, there are no confirmed cases of ovarian adrenal rest tumor, with adrenal rest nodules having been found in the broad ligament and near the ovarian hilus in a 35-year-old woman with 3βHSD deficiency, but not in the ovaries themselves (10).

To date, only two series of 3βHSD deficiency with more than 10 subjects have been reported (11, 12), and there is no large series describing the characteristics of patients with the p.Pro222Gln mutation which is the most frequent mutation encountered in Algeria, being found in all families but one in our series, and is also found in Latin American countries such as Colombia and Brazil (13–15). The hormonal criteria of a high 17OHPreg [basal or ACTH stimulated >90 nmol/l (16)] is preferred to the Δ5/Δ4 ratio [17 OH-Pregnenolone/Cortisol>103 or 181 nmol/l (16, 17)]. With liquid chromatography coupled to the tandem mass spectrometry (LC-MS/MS) method, these cut-offs have yet to be established. Moreover, genetic testing (when available) is recommended to confirm the diagnosis.

In Algeria, a country with a high birth rate of 22.2 births/1,000 population (18) and high levels of consanguinity (38%), we have accumulated a series of 14 patients from 10 families with confirmed 3βHSD2 deficiency (3BHSD2). We have been struck by the relative frequency of the disorder compared with other causes of CAH, how frequently it is misdiagnosed as 21-OHD, and the high rate of sibling deaths in the families. The purpose of this study, therefore, is to detail the presentation and outcome of 3βHSD deficiency in our Algerian families, make an estimate of its prevalence among other forms of CAH, and draw attention to some long-term problems and complications. These include developmental delay, ovarian adrenal rest tumor, and polycystic ovary syndrome (PCOS).

## Patients and Methods

Clinical and hormonal data were collected from the medical records of patients attending a single center, the Pediatric Department of the Centre Hospitalo-Universitaire (CHU) of Bab El Oued, Algiers, Algeria over a fourteen-year period (2007–2021). Although patients from all over Algeria attend CHU Bab El Oued, at least ten other units (pediatric and adult) also receive endocrine referrals. In the absence of a national registry of CAH or rare diseases, and in an attempt to ascertain the exact number of patients followed for 3βHSD2 deficiency during the study period, we contacted all pediatric endocrinologists in Algeria, asking if they had seen one or more confirmed cases. Also, to estimate the prevalence of 3βHSD2 deficiency among other forms of CAH, we compared the number of patients with 3βHSD2 deficiency to the number of patients with other forms of CAH in our department.

### Data Retrieval

Details from the case notes of the patients studied were recorded using an electronic form (Epi-info7) and included the following: date and year of birth, sex, birth weight and gestation, mode of delivery, age at presentation, start of medical treatment, and definitive diagnosis of 3βHSD deficiency. Details of the presence and degree of consanguinity; and a history of sibling deaths from a) salt-wasting (indicative of 3βHSD2 deficiency); and b) unclassified illness during infancy, were recorded. Examination findings including Prader stage (19) and the External Masculinisation Score (EMS) described by Ahmed and colleagues (20) were also recorded. Finally, biochemical and radiological data, and details of surgical and medical treatment were collated.

### Clinical Review

In April 2019, and again in March 2021, all patients were invited to attend CHU Bab El Oued for clinical assessment, which included auxology, expressed according to the 2007 WHO References and standards (21, 22), blood pressure measurement, pubertal staging, Prader and EMS scoring, and clarification (where necessary) concerning consanguinity and sibling health. An IQ test was also performed using the Wechsler scale [Wechsler Preschool and Primary Scales of Intelligence (WPPSI) (23)] and the Khos block-design test (24) for preschool children. Further biochemistry and radiology assessments were also carried out at this time. When patients were fully assessed in both 2019 and 2021, the most recent clinical and biochemical data are given in the *Results* section.

### Biochemistry Assays

Blood samples were normally collected between 8 and 10 a.m. Cortisol, 17-hydroxyprogesterone (17-OHP), serum dehydroepiandrosterone sulfate (DHEA-S), delta4-androstenedione (Δ4A) and testosterone were measured in the laboratory of the department of nuclear medicine in CHU Bab El Oued using radioimmunoassay (RIA). Renin levels were measured in the laboratory of the Centre Pierre Marie Curie Hospital, Algiers, using RIA (Cisbio Bioassays).

Since 17-hydroxypregnenolone (17OHPreg) assay is not available in Algeria, blood samples were sent to Laboratoire Cerba, France and measured using liquid chromatography coupled to tandem Mass Spectrometry LC MS/MS method. Some steroids were reassessed in 2019 and 2021 by LC MS/MS at Lyon University Hospital, France (17OHP, DHEA, 17OHPregnenolone).

Age-appropriate reference ranges are given in the *Results* section and are taken from values established in the laboratory of Lyon, France, supplemented in the case of DHEA by data from Kushnir et al. (25) (please see **Supplementary Table S1**). Normative data from Lyon were determined from plasma samples, drawn at 8 a.m. in subjects beyond early childhood, using the LC MS/MS technique.

### Genetic Analysis

Genetic analysis, after informed consent, was performed at the Department of Molecular Endocrinology and Rare Diseases, Lyon University Hospital, France, as previously described by Sanger sequencing (26) and *in vitro* functional studies (14).

### Ethical Approval

Written informed consent was obtained from all families for genetic testing. The local ethics committee was informed and approved the study as a clinical audit.

### Statistical Analysis

Anthropometric data were expressed as standard deviation score (SDS) using the World Health Organization 2007 data (WHO 2007, Anthro plus software) (21, 22). Data analysis was carried out using the software Epi Info 7 (7.2.2.6). A Student t-test was used to compare the age at diagnosis and treatment in male and female patients.

## Results

**Figure 1:**
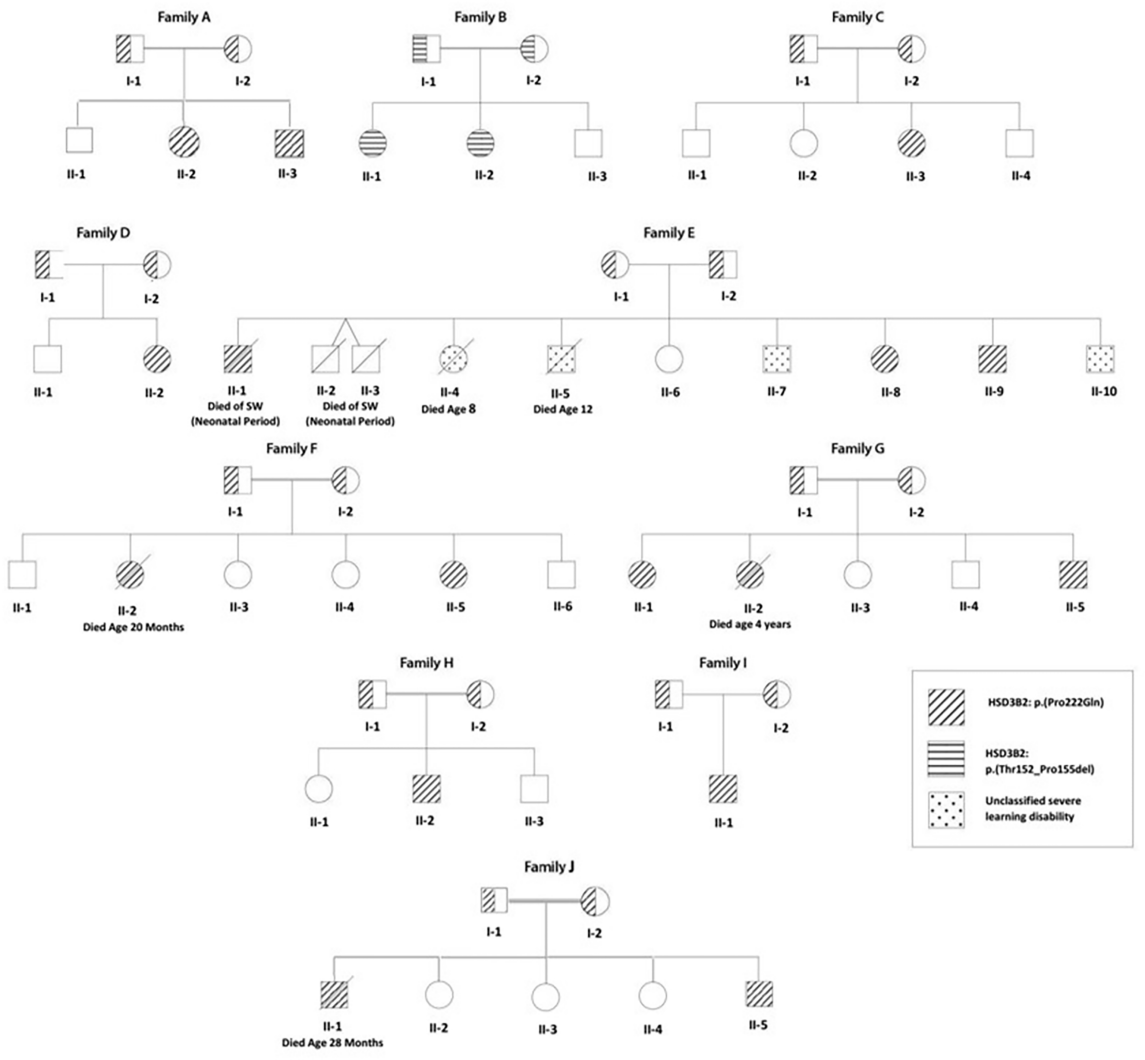
Family tree of 10 Algerian families (8 consanguineous) with a) 3βHSD2 deficiency (individuals shown as hatched circles or squares); and b) an unclassified severe learning disability syndrome (affected individuals shown as speckled circles or squares). A HSD3B2: p.Pro222Gln mutation was found in 9 families (diagonal hatching), while Family B shows a novel HSD3B2: p.Thr152_Pro155del mutation (horizontal hatching). Of 42 children born to the 10 families, 3βHSD2 deficiency was genetically proven in 18 (2 deaths) and suspected on clinical grounds in twins from Family E with neonatal death from salt-wasting.

At the end of the study period, 273 patients from 227 families had been diagnosed with classic CAH in our clinic at CHU Bab El Oued. Of these, 3βHSD2 deficiency was diagnosed and confirmed by molecular studies in 14 patients from 10 families, and their pedigrees are shown in **Figure 1**. After 21-hydroxylase deficiency, with 243 patients from 207 families, 3βHSD2 deficiency was the next most common form of CAH, accounting for 5% of cases, and was more frequent than 11-βhydroxylase deficiency (13 patients from 8 families) and StAR protein deficiency (6 patients from 4 families).

The fourteen patients (eight females) were from ten families, with consanguinity (parents first cousins) in eight. Four patients from three families (F, G, and I) were from the same region in the north-center of Algeria, the province of Boumerdès (**Figure 1**).

Four children (3 boys) from family E, a family with poor socioeconomic circumstances, suffered from a separate severe congenital motor disability syndrome. Two of these children, E II-4 and II-5, died at the ages of 8 and 12 years with severe malnutrition.

Six siblings from 4 families died in infancy, of whom three (Family E II-1, II-2, and II-3) had a clear history of salt-wasting, while three (Family F II-2, G II-2, and J II-1) died with adrenal insufficiency while on hydrocortisone treatment. The median (range) age at death for these six siblings was 19.4 (0.5–48) months.

**Table 1** shows the clinical features of the 14 patients with confirmed 3βHSD2 deficiency. Four patients had been diagnosed originally as having 21-OH deficiency (B II-1; B II-2; E II-8; and G II-1) and were treated with hydrocortisone only; two patients (A II-3 and B II-2) were diagnosed soon after birth by screening since one sibling was already being managed for CAH (A II-2 and B II-1). Patient B II-2 was screened at birth, but with suspected 21OHD.

**Table 1:** Clinical data and features at first examination for 14 Algerian patients from 10 families with confirmed 3β-hydroxysteroid dehydrogenase (3βHSD) deficiency.

Patient No.	Pedigree	Parental consanguinity	Sex	BW (kg)	GA (w)	Mode of presentation	Genital status at diagnosis		SW	Age at presentation	Age at start of treatment	Age at definitive diagnosis of 3βHSD	Genetic mutation
							Females Prader stage (Clitoral length in cm)	Males EMS (Penile length in cm)					
**1**	A II-2	1st cousin	F	3.35	37	SW	1 (ND)	–	+	3w	7w	7w	p.(Pro222Gln)
**2**	A II-3	1st cousin	M	3.25	39	DSD + SCR	–	3.5 (2)	+	3 d	3 d**	3 d	p.(Pro222Gln)
**3**	B II-1	1st cousin	F	4	41	SW + DSD	2 (4)	–	+	3m	4m*	6w	p.(Thr152_Pro155del)
**4**	B II-2	1st cousin	F	2.7	41	SCR	1 (0.5)	–	+	4w	1m *	5w	p.(Thr152_Pro155del)
**5**	C II-3	2nd cousin	F	3.2	41	SW + DSD	2 (1.5)	–	+	13m	13m	16m	p.(Pro222Gln)
**6**	D II-1	No	F	2.6	41	SW	1 (ND)	–	+	14d	6w	6w	p.(Pro222Gln)
**7**	E II-8	2nd cousin	F	ND	41	SW	2 (1.5)	–	+	14d	4w*	5w	p.(Pro222Gln)
**8**	E II-9	2nd cousin	M	3	40	SW + DSD	–	6 (2)	+	19d	3w	5.3m	p.(Pro222Gln)
**9**	F II-1	1st cousin	F	ND	41	SW	2 (1)	–	+	17d	3m	7.3m	p.(Pro222Gln)
**10**	G II-5	2nd cousin	M	5	41.5	SW + DSD	–	9 (3)	+	3d	3d	3d	p.(Pro222Gln)
**11**	G II-1	2nd cousin	F	3.4	41	SW + DSD	2 (ND)	–	+	14d	1m*	15y	p.(Pro222Gln)
**12**	H II-2	1st cousin	M	4	41	SW	–	6 (2)	+	2m	2m	3.8m	p.(Pro222Gln)
**13**	I II-1	No	M	3.3	40	DSD		6 (2)	+	3w	3w	3w	p.(Pro222Gln)
**14**	J II-5	2nd cousin	M	3.7	40	DSD + SW		3 (2.8)	+	4m	4m	16m	p.(Pro222Gln)

Age at presentation, start of treatment and definitive diagnosis of 3βHSD deficiency is given in days (d), weeks (w), months (m) or years (y). BW, birth weight; GA, gestational age; SW, salt-wasting; F, female; M, male; EMS, External Masculinization score (maximum 12); SCR, screening; ND, not documented; DSD, disorder of sex development. *Initially diagnosed as 21-OH deficiency. **Treatment was started at birth, the patient presented with SW subsequently.

### Prevalence of 3βHSD Deficiency

Apart from the 14 confirmed and six unconfirmed but probable patients mentioned, we are aware of only two other patients with 3βHSD, one diagnosed biochemically in our center, in whom genetic studies are pending, and the other being followed by a colleague in France. However, since children are also sometimes followed by adult endocrinologists and other children have probably died in infancy, this number is almost certainly an underestimate.

### Presentation of the 14 Confirmed Patients

(See **Table 1**) In the absence of any systematic newborn screening program in our country, all but two patients (A.II.3 and B II-2), who were diagnosed by neonatal family screening, presented with severe salt-wasting (SW) during infancy, mean ± SD (range) age 2.2 ± 3.3 (0.1–13) months. SW syndrome was associated with a disorder of sex development (DSD) in all male patients, but was not the principal cause of referral. Two patients presented with SW in the early neonatal period (3–10 days), 7 aged 11–28 days, and 5 after 28 days.

The median (range) age at presentation with either SW, DSD or both was 2.4 weeks (3 days–13 months). There was no male predominance in our patients, despite the absence of ambiguous genitalia in females. Mean ± SD age at clinical/biochemical diagnosis was 1.3 ± 1.5 months in males and 2.4 ± 4.3 months in females (p = 0.5).

The median (range) age at the start of treatment with hydrocortisone was 1.25 (0.1–13) months. Since fludrocortisone is not widely available in Algeria, mineralocorticoid treatment was not always possible and was often not administered regularly.

The median (range) age of the patients at the time of referral to our department at CHU Bab El Oued for further investigations was 50.5 months (3 days–16.5 years). Ten were seen within the first year of life, while 4 females (B II-1, B II-2, E II-8, and G II-1) were referred after the age of 10 years (10.4–16.5 years). These four patients were already receiving steroid treatment and had been misdiagnosed as having 21 OHD.

### Presentation, DSD Status and Definitive Diagnosis in Females

**Figure 2:**
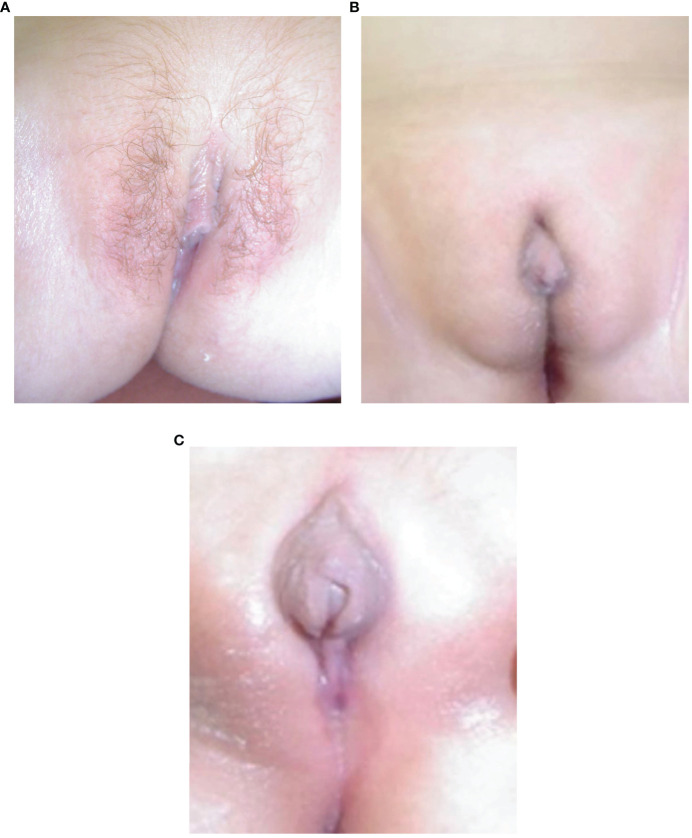
**(A–C)** Appearance of external genitalia in two siblings from family E with 3βHSD2 deficiency due to a p.P222Q mutation, showing virilization with clitoromegaly and pubic hair in the sister, E II-8 **(A)** and under-masculinisation in the brother, EII-9 **(B, C)**.

The eight females presented with salt-wasting only (5), salt-wasting with clitoromegaly (2), and after being screened at birth (1). Virilization in girls was mild, with two patients not significantly virilized, two at Prader stage 1 (clitoromegaly only), and 4 at Prader stage 2 (clitoromegaly with narrowing of the distal vagina) (see **Figure 2A**). None had labial fusion. Clitoromegaly was more severe (4 cm) in patient E II-8, in whom the diagnosis was made well after the neonatal period at 3 months (**Figure 2A**). At presentation at 13 months, one girl (C.II.3) had Prader-stage P2 pubic hair.

Due to non-availability of fludrocortisone, the four older female patients who had been initially misdiagnosed as 21-OH deficiency and had been treated with hydrocortisone alone. In these patients, adjustments to hydrocortisone dosing had been made in relation to 17OHP levels and not to 17OHPreg levels, leading to inadequate treatment.

### Presentation, DSD Status and DSD Management in Males

The six males presented following family screening (1), with genital anomaly (1), salt-wasting (1), and both genital anomalies and salt-wasting (3). Two males were severely under-masculinized with EMS scores of 3 and 3.5/12, including patient E II-9 (**Figures 2B, C**) and two mildly under-masculinized (EMS scores of 6 and 9/12), including patient G II-5. All six patients received testosterone enanthate (50 mg/month for 3 months) during the first months of life, and four underwent uncomplicated surgical correction of hypospadias. So far, one patient (E II-9) has developed spontaneous puberty without any need for testosterone supplementation.

### Biochemical Data

**Table 2** shows the initial and current biochemical status of the 14 patients with confirmed 3βHSD deficiency. The sensitivity of the hormones measured in showing values above the reference range was 100% for 17 OH-pregnenolone and DHEA-S except in patients in whom the measurements were obtained while on treatment.

**Table 2:** Hormonal data in 14 Algerian patients with 3β-HSD deficiency.

	First available analysis						Last available analysis (LC-MS/MS)			
Patient No.	17OHP (nmol/l)RIA	Delta4-A (ng/dl)RIA	17 OH-Preg (nmol/l)(LC MS/MS)	DHEA-S (µg/dl)RIA	ACTH (pg/ml)	Renin (pg/ml)	17OHP LC-MS/MS (nmol/l)	17 OH-Preg LC-MS/MS (nmol/l)	DHEA LC-MS/MS (nmol/l)	DHEA-S LC-MS/MS (nmol/l)
1/A II-2	60	6.06	140.4	464.38		933	4.12	119.69	30.60	4.82
2/A II-3	0.37*			9.4*		2*	<0.3*	1.06*	0.89*	
3/B II-1	164.3	5.73	1297	900	2,135	851	2.14			
4/B II-2	320	2.34	20.8*	1,000	133	960	1.47			
5/C II-3	242	2.24	112.21	538	475	360	4.3	67.57	30.60	3.98
6/D II-1	19.26*	1	159.33	1,105	30.98	1,040	3.5	131.99	77.28	2.88
7/E II-8	7.75*	0.98	127	120		597	1.2	27.65	77.08	
8/E II-9	99	4.95	139	5,441.29		540	7.3	92.63		
9/F II-1	84.85	0.32	295	150			1	43.02	3.46	
10/G II-5	1.83*	2.33*	17.75*	1,080*		16,634	3.5	89.13	27.62	16.63
11/G II-1	804		157	687		10,665	2.14	93	4.37*	20.14
12/H II-2	60	0.01	10.9*	4.32	234.8	802	4.15	127.2		19.35
13/I II-1	73.7		1,500	53			0.08*	1.75*	0.30*	2.9*
14/J II-5	41.6			34.84			3.23*			
Reference Range	[0.4–3.3]	[0.21–3.08]	[0.13–13.7]	[30–333]	[29–38]	[360–1,040]	[0.49–1.87]	[0.13–13.7]	0.5–2 y [0.2–8.7]	1–4 y: [10–530] 5–9 y [80–2,310]

^*^Analysis performed while on hydrocortisone treatment. 17-OHP, 17 hydroxyprogesterone; 17OH-Preg, 17 hydroxypregnenolone; DHEAS, dehydroepiandrosterone sulfate; ACTH, adrenocorticotrophic hormone; Delta4-A, Delta 4-Androstenedione; LC-MS/MS, Liquid Chromatography coupled to tandem Mass Spectrometry.

Please see **Supplementary Table S1** for age-appropriate reference ranges. Hormonal analysis was performed where possible either before starting treatment or within a day of treatment.

Initial 17OH-Progesterone (17OHP) was mildly elevated at 79.2 (7.7–804) nmol/l) [normal values 0.4–3.3], while 17 OH-Pregnenolone (17OHPreg), DHEA-S and renin were elevated in all patients, respectively—157 (112.2–1500) nmol/l for 17OHPreg [normal values 0.13–13.7]; 687 (53–5442) µg/dl for DHEA-S [30–333]; and 892 (360–16,634) pg/ml for Renin [360–1,040]. Delta4-Androstenedione was only mildly elevated in some patients (2.24 (0.01–6.06) ng/dl [normal values 0.21–3.08]. When reassessed by LC-MS/MS (patients off treatment for one day), 17OH-Preg was high in most patients at 89.13 (1.06–132) nmol/l, while 17 OHP [2.7 (0.08–7.3) nmol/l] and DHEA-S [4.82 (2.88–20.14) nmol/l], were normal or only slightly elevated in all patients.

### Genetic Analysis

**Figure 3:**
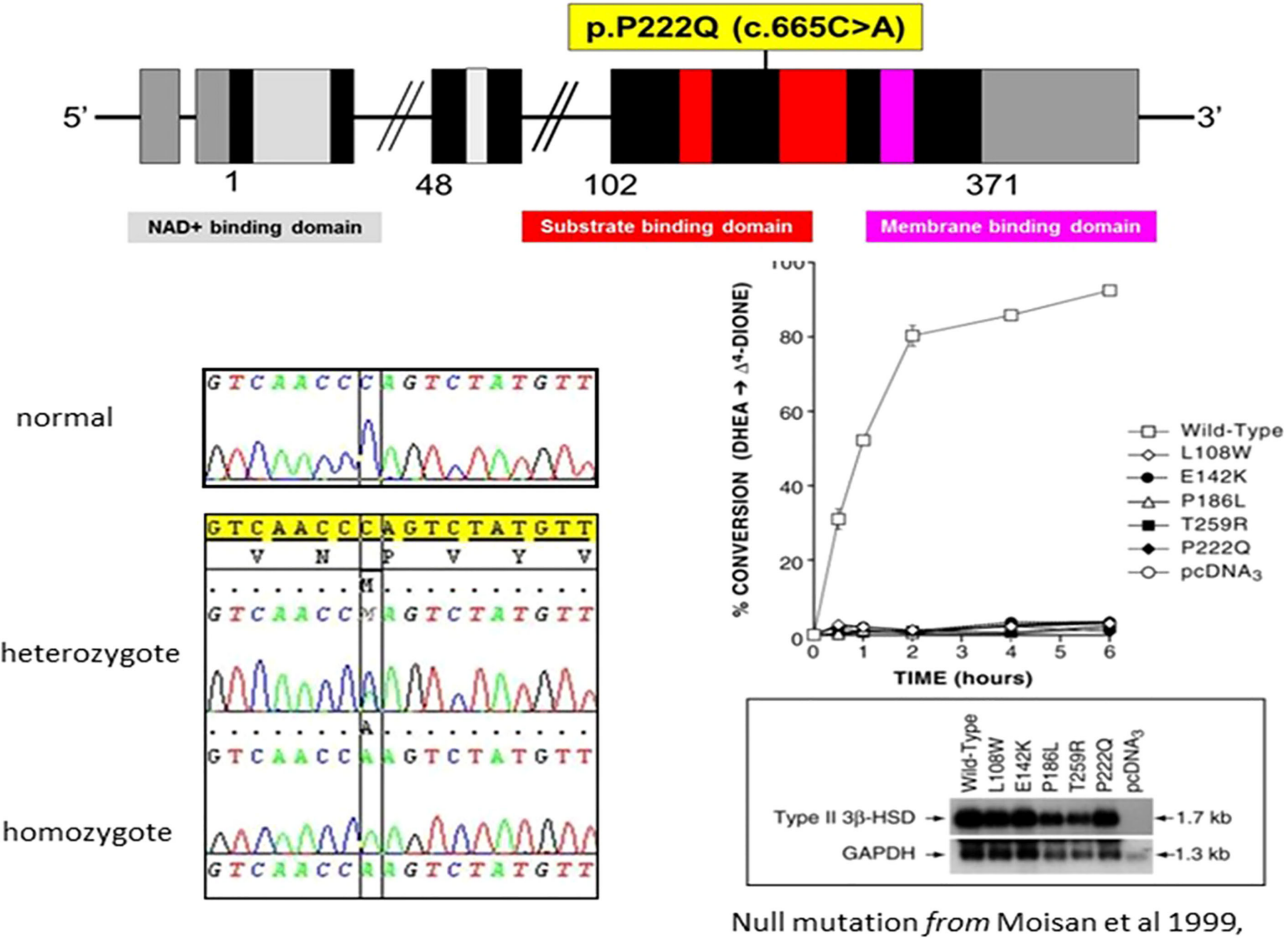
Characteristics of the p.P222Q mutation of the HSD3B2 gene [From Moisan et al, 1999 (27)] —reproduced by kind permission of Oxford University Press).

(See **Figures 1**, **3 ** and **Table 1**) All but two of the 14 patients were homozygous for the null mutation, p.(Pro222Gln) (c.665C >A). The two sisters of Family B were homozygous for a novel 12bp deletion (c.453_464del) deleting 4 amino acids p.(Thr152_Pro155del). As these amino acids are located within the characteristic catalytic Y-X-X-X-K site, this mutation should be a null mutation, hence the good genotype/phenotype correlation observed (**Figure 3**).

### Clinical Outcomes

**Table 3** shows the status of the 14 patients at the last review in 2019 or 2021. All patients were treated with hydrocortisone at a mean (± SD) dose of 15.2 ± 0.8 mg/m²/day. Owing to problems with fludrocortisone availability, three patients were not receiving this at the time of their last evaluation, and the remaining patients were on a dose of 54 ± 25 µg/day. Of note, fludrocortisone treatment is either imported from Spain twice a year in bulk by compassionate health professionals or provided at cost or for free to the patients at the discretion of the pediatric endocrinologist or shipped directly by family members living abroad (28).

**Table 3:** Status at most recent follow-up in 14 Algerian patients with genetically confirmed 3β-hydroxysteroid dehydrogenase.

Patient No. Pedigree	Sex	Age (yr)	BA(yr)	BP (mmHg)	HC dose (mg/m²/d)	FC dose (µg/d)	Height (cm/SDS)	BMI (kg/m²)/SDS	Tanner Stage	Age at B2/G2	Age at P2	Age at menarche	Complication	IQ/DQ
1/A II-2	F	11.5	12	95/60	16.2	25	150/+0.31	16.4/−0.54	B3P4A3	8.5	5	–	Premature pubarche	78
2/A II-3	M	1.67	ND	80/50	15	50	79/−1.96	18.3/+1.67	G1P1A1	–	–	–	Short stature	ND
3/B II-1	F	18.32	>18	115/75	15	100	157/−0.93	24.7/+0.97	B4P4A3	9	10	11.5	PCOS Adrenal tumor	105
4/B II-2	F	17.75	18	110/75	15	100	151/−1.81	19.7/−0.53	B4P4A3	11	10	14.5	GH treatment for short stature PCOS Adrenal tumor	99
5/C II-3	F	8.75	8.83	90/60	15.9	50	128/−0.53	25/−0.58	B2P3A1	8.5	1.25	–	Premature pubarche	98
6/D II-1	F	8	9	90/60	14.8	50	133/+1.06	37.7/+2.21	B2P3A1	8	5.5	–	Premature pubarche Obesity	87
7/E II-8	F	18.32	17	90/70	14.5	*	151/−1.8	18.4/−1.09	B4P5A3	11	10	14	Learning disability Probable OART	43
8/E II-.9	M	14.32	14	100/60	14.38	*	161/−1.15	16.6/−1.36	G4P5A3	11.5	7	–	TART Learning disability	49
9/F II-1	F	8.22	7.83	90/40	15	50	120.5/−1.25	20/+1.77	B1P1A1	–	–	–	–	80
10/G II-5	M	6.7	7	80/60	15	50	127.5/+1.45	17.2/+1.15	G1P1A1	–	–	–	TART	90
11/G II-1	F	21.7	>18	90/70	16;2	50	165/+0.28	27.5/+1.58	B5P5A3	13	10	14.5	PCOS Adrenal tumor	104
12/H II-2	M	9	12	90/70	14	*	136/+0.549	24.9/+311	G1P2A1	–	8	–	Obesity Learning disability	55
13/I II-1	M	4.37	4	80/60	13.5	25	106/+0.05	25.8/+6.3	G1P1A1	–	–	–	Obesity	98
14/J II-5	M	4.7	4	90/65	16.5	50	110/−0.37	14/−0.95	G1P1A1	–	–	–	–	90

BA, bone age; BP, Blood Pressure; HC, hydrocortisone; FC, fludrocortisone; BMI, body mass index; PCOS, polycystic ovary syndrome; TART, testicular adrenal rest tumor; OART, ovarian adrenal rest tumor; IQ, Intellectual quotient; DQ, developmental quotient (in children aged <3 years); NA, not available; ND, not done (not appropriate for age); ^*^ FC stopped due to lack of availability. IQ could not be done but the child had bad results at school.

At the most recent visit, the median age was 8.7 (1.7–21.7) years, height 0.24 (−1.96 to +1.45) SDS, with 5 patients <−1 SDS; BMI +1.06 (−1.36 to +6.3) SDS, with 7 patients >+1 SDS and 3 patients >+2 SDS.

Seven girls reached Tanner B2 and P2 during the study period, at 9 (8–13) and 10 (1.25–10) years old. Only one boy (E II-9) had entered puberty at G2 aged 11.5 years. In the absence of adequate treatment, this patient had already presented with premature pubarche aged 7 years. Another boy (H II-2) presented with premature pubarche at the age of 8 years.

### Complications of 3βHSD Deficiency

Six of the 14 patients experienced one or more acute illnesses with SW crises after diagnosis, but there were no deaths.

Overweight (BMI >1 SDS) was seen in seven patients. Only three patients were obese (BMI >+2 SDS) even though all subjects were receiving hydrocortisone doses that were above the physiologic replacement level of 8 mg/m^2^/day. However, we were unable to demonstrate a direct relationship between obesity and hydrocortisone dose, which was between 13 and 14.8 mg/m^2^/day in the three obese patients.

Although the four girls reaching menarche during the study period experienced this within the normal age range (11.5–14.5 years), three of these girls (patients B II-1, B II-2, and G II-1) had oligo-amenorrhea and met the criteria for PCOS (29) with a combination of menstrual irregularity, clinical features of hyperandrogenism (hirsutism and severe acne), and enlarged, cystic ovaries. Ovarian volumes were very large in all three girls: 73 × 47 × 40 mm and 54 × 40 × 30 with cysts up to 68 × 40 mm in B II-1; 54 × 20 × 30 and 63 × 30 × 20 with cysts >25–35 mm in B II-2; and 48 × 42 × 55 and 84 × 55 × 40 mm with cysts >40 mm in G II-1. Patient E II-8 also had large ovaries (29 × 28 × 49 and 33 × 22.5 × 39) with large cysts measuring 38 × 36 mm on the most recent pelvic ultrasound. However, this girl did not have either prolonged amenorrhea or severe hyperandrogenism, and so the diagnosis was one of the polycystic ovaries rather than PCOS.

Stature was normal, although one patient had received growth hormone therapy to offset short stature with bone age advance.

### Adrenal Tumor Formation, Testicular Adrenal Rest Tumor and Ovarian Adrenal Rest Tumor

Two male patients (E II-9 and G II-5) were diagnosed with testicular adrenal rest tumor (TART) by systematic testicular ultrasonography at 5 and 10 years, testicular examination having revealed no abnormality. One patient (E II-9) had been inadequately treated during infancy and childhood because of fludrocortisone unavailability and poor compliance.

**Figure 4:**
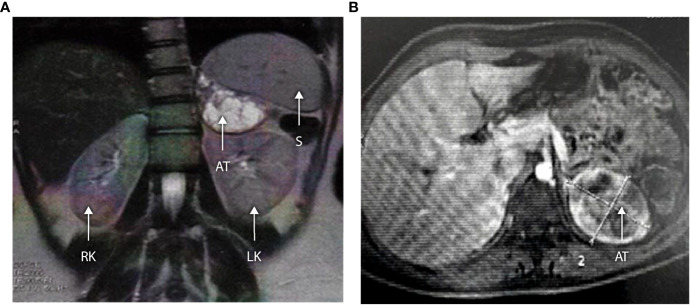
**(A, B)** Abdominal MRI scan in a 16-year-old with 3βHSD2 deficiency (Patient B II-1). Axial and coronal sections demonstrate a large left-sided adrenal tumor measuring 63 × 52 × 51 mm. The lesion shows central cystic degeneration and is pushing the kidney downwards. RK, right kidney; LK, left kidney; S, spleen; AT, adrenal tumor.

**Figure 5:**
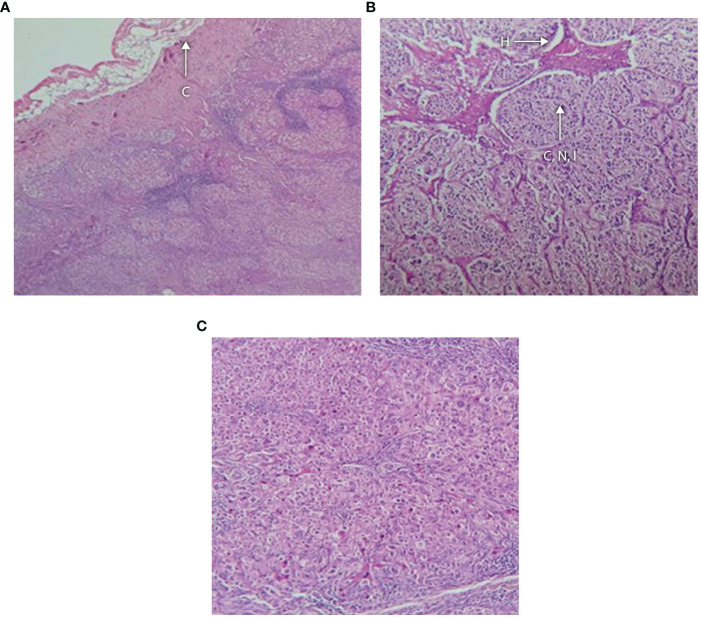
**(A–C)** Histology of adrenal tumor from patient G II-1 following surgical removal showing **(A)** fibrous capsule with an underlying neoplasm containing hemorrhagic foci, no vascular or capsular invasion; **(B)** tumor composed of cells arranged in nests and cords separated by vasculature and lymphoid tissue; and **(C)** higher magnification showing that the cells have distinct boundaries and clear cytoplasm with monomorphic nuclei and foci of oncocytic metaplasia. There is hyperchromasia of the nuclei and apoptosis. C, capsule; H, hemorrhagic focus; CNI, cords, nests and islands of tumor.

The three older girls (B II-1, B II-2, and G II-1) with PCOS also presented with adrenal masses at 13, 15, and 16 years of age (see **Table 3** and patient B II-1 in **Figure 4**). In patient G II-1, routine pelvic ultrasonography showed a large right adrenal mass, measuring 27 × 30 mm. This mass was of suspect appearance on pelvic computed tomography with heterogeneous enhancement, including necrotic areas in contact with the inferior vena cava, and was therefore surgically removed and analyzed in view of the suspicion of malignancy. Initial pathological analysis favored an adrenocortical tumor. After a second analysis, the diagnosis was revised to adrenal cortical hyperplasia secondary to under-suppressed CAH (**Figure 5**). Post-operatively, hyperandrogenism persisted in this patient, and pelvic computed tomography revealed a large solid mass measuring 40 × 42 mm within the left ovary, which was polycystic as described above. This finding was considered highly suggestive of an ovarian adrenal rest tumor (OART).

Systematic pelvic ultrasonography also showed adrenal masses in the two affected sisters of family B. The older sister (B II-1) was 15 when the mass was diagnosed, a large left adrenal mass measuring 63 × 52 × 51 mm (see **Figures 4A, B**). The evaluation showed no clinical, biological, or radiological evidence of pheochromocytoma. The adrenal mass was removed, and the analysis favored adrenal cortical hyperplasia. Her sister (B II-II) had a left adrenal mass measuring 20 × 25 mm which is currently being kept under surveillance.

### Intelligence Quotient

(see **Table 3**, Far Right-Hand Column) An intelligence quotient was assessed in all but one patient, who was too young to be tested. The median IQ (range) was 90 (43–109) (n = 13) and the scores were ranked as follows: 100–110, n = 2; 90–99, n = 5; 80–89, n = 2; 70–79, n = 1; <70, n = 3. The three patients with IQ scores <70 were H II-2 (IQ 55), E II-9 (IQ 49), and E II-8 (IQ 43). Of note, siblings E II-8 and 9 are from kinship in which other siblings had an unclassified global neuro-disability disorder featuring severe cerebral palsy, which appears unrelated to 3βHSD2 deficiency. However, both parents of family E and their one unaffected child (E II-6) are of normal intelligence. There was no correlation between IQ and age at the start of treatment (p = 1).

## Discussion

### Prevalence

Despite the impossibility of establishing the exact prevalence of 3βHSD2 deficiency, given the absence of a national program of neonatal screening and the lack of national registries for rare diseases in the Maghreb countries, we have nevertheless observed that the prevalence of 3βHSD2 deficiency appears higher in Algeria than elsewhere. Indeed, in a large cohort including all patients with defects in steroid biosynthesis investigated in the laboratory of molecular endocrinology and rare diseases of Lyon Hospital, France, 3βHSD2 deficiency is the most rare form of CAH (2). Globally, 3βHSD2 deficiency is estimated to account for less than 5% of all CAH and is extremely rare except in specific populations such as the Old Order Amish in North America (11) and Turkey (12). Even so, the prevalence described in our series is certain to be an underestimate because of patients dying undiagnosed and the misdiagnosis of 3βHSD as 21-OHD. In countries with neonatal screening programs for 21OHD, patients with 3βHSD2 deficiency may be detected at birth (30). In the absence of such a program, diagnosis depends on clinical awareness, as discussed below.

### Diagnosis

Diagnosis in a salt-wasting under-masculinized male is easy, but paradoxically difficult in females who are more likely to die undiagnosed with salt wasting (31). This situation, in which girls die undiagnosed with 3βHSD2 deficiency is to be compared to boys with 21-OHD who die undiagnosed.

The p.Pro222Gln mutation of the HSD3B2 gene is one of the most frequent severe mutations and is predominant in the Algerian population. It has also been found in Colombia and Brazil (13, 15), probably due to a founder effect (2). Although this mutation is described as severe with severe SW forms, one of our patients was diagnosed at 13 months with a delayed SW presentation, clitoromegaly, and premature pubarche. This observation, in contrast with those of patients presenting very early with SW, illustrates the phenotypic variability that may occur with the same genetic defect, although there is usually a good genotype/phenotype correlation. This discrepancy could be explained by the presence of other possible mutations in non-explored genes involved in steroidogenesis in a consanguineous family.

The biochemical diagnosis of 3βHSD2 deficiency is based on the elevation of Δ5-steroids (17 OHPreg, DHEA-S) compared to Δ4 steroids [(17 OHP, Delta4-Androstenedione)]. Because of the conversion of 17OH-pregnenolone to 17 OH-progesterone by the 3βHSD 1 enzyme in peripheral tissues, 17 OHP levels may be increased, leading to the misdiagnosis of 3βHSD2 deficiency as 21OHD (31). We have observed that 17 OHP was mildly elevated in our patients compared to 17 OHPreg. Unfortunately, the 17 OHPreg assay is not widely available in Algeria and is only available in specialist laboratories, which therefore necessitates sending blood samples abroad—a measure that is costly and too expensive for some families.

Therefore, in the absence of available and affordable analysis of 17OH-pregnenolone, and any newborn screening program, clinicians should consider the diagnosis of 3βHSD2 deficiency in all under-masculinized boys and non-virilized or slightly virilized girls who present with mildly elevated 17OHP, elevated ACTH, and SW with elevated renin.

The elevation of 17-OHP on RIA observed in this series is of potential interest regarding newborn screening for CAH. After excluding four patients who were already receiving steroid treatment, the initial 17-OHP values in the remaining 10 patients were all above the French threshold of ≥17 nmol/L for infants ≥36 weeks of gestation (32). By contrast, when using the 17OHP–LC-MS/MS method, all values were well below this cut-off, the difference being attributable to cross-reaction with other steroids when the immunometric assay is used. At present, newborn screening techniques are usually immunological and cross-react with 17-OH pregnenolone, so that 3βHDS2 deficiency would be expected to be detectable. However, if these immunological techniques were to be replaced by LC-MS/MS (which has the advantage of reducing false positive tests and the significant cost they generate), 3βHDS2 deficiency might not be detected. Therefore, if newborn screening for CAH was established in Algeria and other Maghreb countries in the future, an immunological technique combined with current French thresholds would be preferable, to detect both 3βHSD2 and 21-OH deficiency.

The diagnosis of 3βHSD2 deficiency should always be confirmed by 17-OHPregnenolone measurement and by genetic analysis in countries where it is available.

### Outcomes

Unlike 21-OH deficiency, very few studies have described the outcomes of patients with 3βHSD2 deficiency (see **Table 4**) and most have focused on male patients.

**Table 4:** Studies showing outcomes in patients with 3-β hydroxysteroid dehydrogenase 2 deficiency.

First Author/year of publication (Reference)	Country/Ethnicity	Sex	Mutation	Complication/Puberty/gonadal status
Parks/1971 (33)	USA	M [1]	W171X	Acne 11 years, pubic hair and gynecomastia at 12 years
Jänne/1974 (34)	Finland	M [1]		Premature pubarche, gynecomastia. Testosterone gel started at 9 years Normal testicular histology
Schneider/1975 (35)	USA	M [1]		Onset of puberty at 10 years, gynecomastia aged 11 years with acne, obesity Immature testis, predominantly Sertoli cells, Leydig cell hyperplasia, spermatogenic arrest
Zachman/1979 (36)	Switzerland	F [1]		Severe salt wasting crises during infancy, normal mental development Bone age delay; puberty induced. Adult height 159.5 cm
Martin/1980	Finland	M [1]		Obesity, gynecomastia
Mendonca/1987 (15) Moisan/1999 (14)	Brazil	M [1]	A82T	46, XY individual, initially raised as a girl, virilization during puberty; changed gender at 17 years Gonadectomy and penile surgery at 7 years; Induced puberty; normal testicular histology
Rheaume/1992 (27)	Switzerland USA	F [1] M [1]	W171X W171X/186insC-fs	Lack of spontaneous breast development, virilization Spontaneous puberty at 13 years, gynecomastia; normal spermatogenesis Fathered two children (but no genetic confirmation)
Chang/1993 (37) Moisan/1999 (14)	USA	F [1] M [1]	G129r/c6651G>A	Breast development 10 years, menarche at 12 years; adult height 158 cm; irregular menses, hirsutism; bilateral enlarged ovaries, multiple cysts (PCOS) Androgen excess, advanced bone age
Yoshimoto/1997 (38)	Japan	M [1]	R249X	Gynecomastia at 7.5 years, Normal pubertal development; no mature spermatogenesis
Alos/2000 (39) Moisan/1999 (14)	French Canadian	F [1] M [1]	A10E	Advanced puberty and bone age at 8 years. Menarche at 10.3 y; enlarged ovaries with multiple cysts Pubic hair at 10 years; G2 at 10.5 years; TART; azoospermia
BinAbbas/2004 (40)	Saudi Arabia	M [1] F [1]		Normal puberty; adult height 155 cm; normal sperm count Normal puberty at 14 years, adult height 150 cm; mild hirsutism, menstrual irregularities.
Burckhardt/2015 (41)	Canada/Sri Lanka	M [1]	c.687del27	Cerebral palsy, psychomotor retardation, dyskinetic movement disorder Normal puberty; gynecomastia; spermatogenic arrest (Sertoli cells only)
Lolis/2018 (42)	Sweden	M [1]	Cys-72-Arg	Cryptorchidism. Spontaneous puberty with advanced bone age. Extensive bilateral TARTs from 13 years, mimicking Leydig cell tumor; azoospermia. Adult height 174.5 cm (−2 DS/TH). Cushingoid with obesity and osteoporosis
Falhammar/2012 (43)	Sweden	M [1]		TART, azoospermia
Donadille/2018 (1)	France	M [1]	687 del27	Normal puberty; normal sperm count; adult height 170 cm
Benkert/2015 (11)	USA/Amish	M (2), F (3)	c.35G>A	TART (2 M), PCOS with irregular menses (2 F), obesity (5), early puberty [4] with advanced bone age, hirsutism/acne (5), ischemic encephalopathy (1)
Guran/2020 (12)	Turkey	F [5] M [9]	p.N323D, p.S218P p.W355R	Premature pubarche (F = 5), non-progressive precocious puberty (1 F); central precocious puberty (2F), menarche at 12 years (2F), PCOS (1 F) Premature pubarche (M = 9), non-progressive precocious puberty (2 M), Tanner G5 (3 M) at 14.6, 15.6, and 17 (partial gonadal insufficiency), TART (2 M)
Ladjouze/2022 (44)	Algeria	F [8] M [6]	p.Pro222Gln	Premature pubarche (3 F), menarche at a normal age (4 F), ART (3 F), OART (1 F), PCOS (3 F), Obesity (1 F) Premature pubarche (2 M), spontaneous puberty (1 M). TART (2 M), learning disability (2 M), obesity (2 M)

FH, Final Height; TART, Testicular adrenal rest tumor; ART, Adrenal rest tumor, OART, Ovarian adrenal rest tumor; PCOS, Polycystic ovary syndrome.

Most of the male patients with 3βHSD2 deficiency described in the literature have entered puberty spontaneously (1, 11, 12, 27, 33, 35, 39–42), probably because of the peripheral conversion of DHEA-S to testosterone (41). At present, only one male patient in our study has reached puberty at a normal age, the others being currently of prepubertal age.

Previous case reports have reported a relative frequency of gynecomastia (27, 33, 34, 38, 41, 45, 46) in boys with 3βHSD2 deficiency, attributed to the conversion of the large number of androgen precursors to androstenedione and testosterone by HSD3B1, with these latter hormones being then converted to estrogens with the help of HSD17B1, HSD17B5, and CYP19A1 (41). However, this problem was not reported in the larger case series (11, 12).

Two of the six males in our patients have developed premature pubic hair. Guran (12) and Benkert (11) have reported a high prevalence of premature pubarche and precocious puberty in their patients, despite hydrocortisone treatment. This may be attributed to the increased expression of 3βHSD1, which increases testosterone and Δ4 steroid concentrations in extra-gonadal and extra-adrenal tissues as children mature (12).

Despite the spontaneous development of puberty in most of the male patients, some needed testosterone treatment. Azoospermia (39, 42, 43) was reported in pubertal or adults patients and testicular anatomy was abnormal in some patients, with immature histology. As with 21 OHD, TARTs were frequently reported in male patients, due to sub-optimal treatment (11, 12).

The association of TARTs, incomplete gonadal maturation, and pathological testicular histology are likely to have a negative impact on the fertility of patients with 3βHSD2 deficiency, although this area is not yet well documented (1, 41). However, some patients have shown normal gonadal development with normal testis histology and, normal sperm count (15, 40). One patient was also reported as having fathered two children, although there was no genetic confirmation of 3βHSD2 deficiency in this case (27).

Few studies have evaluated puberty in female patients with 3βHSD2 deficiency (11, 12, 27, 36, 37, 39, 40). In our study, all female patients at an appropriate age had reached puberty spontaneously and had their menarche at a normal age, consistent with reports in the literature. However, we are struck by the relative frequency of premature pubarche in our patients. Indeed, similar to male patients, and probably for the same reasons, premature pubarche and precocious puberty have been reported in female patients with 3βHSD2 deficiency (11, 12). PCOS was also evident in three girls in our series, with polycystic ovaries but not PCOS in a fourth. PCOS has already been described in female patients with 3βHSD2 deficiency (11, 12, 37) as a probable effect of androgen overproduction.

Adrenal tumors have been reported in inadequately treated patients with 21OH deficiency, but not to date in patients with 3βHSD2 deficiency. They are a consequence of chronic elevation of ACTH that leads to adrenal cortical hyperplasia in patients with suboptimal hydrocortisone treatment. In our series, we have been surprised by the discovery, on systematic ultrasonography evaluation, of voluminous adrenal tumors in two female patients. Both had been treated since early infancy and were initially misdiagnosed as 21OH deficiency. Because of this misdiagnosis, the treatment was inadequate; the physicians titrating the hydrocortisone dose according to 17OHP and not to 17OHPreg. Both had very large adrenal tumors that led to surgical removal. One of the tumors was large and presented radiologically and histologically as an adrenocortical tumor. Further histological analysis and the benign evolution of the case allowed the correct final diagnosis to be made.

Unlike TART, OART is rarely described in the CAH literature. As mentioned above, only one publication describes adrenal rest tissue in a woman with 3βHSD2 deficiency (10) but in this case the nodules were adjacent to, rather than within, the ovaries. OART was considered highly likely in one girl in our series (G II-1) who had both PCOS and had also undergone removal of adrenal mass. However, in the absence of histological confirmation, the diagnosis of OART in this girl remains unproven.

Growth patterns in our patients were normal, despite the relatively high doses of hydrocortisone used during some periods because of the problems with mineralocorticoid availability. One patient in our series had short stature and was treated with growth hormone therapy. The few patients who reached final height (FH) had a normal height compared to the WHO references. Few studies report final height in patients with 3βHSD2 deficiency. Normal final height was reported in well-treated patients (1), but FH may be compromised when treatment is suboptimal (36, 37, 40, 42).

Median IQ (range) was in the lower half of the normal range in all but two patients in our series, with subnormal IQ (<70) in three patients, two of which were from the same family (E) in which there is an additional neuro-disability disorder. Given that both the parents and an unaffected sister (E II-6) of this family are of normal intelligence, indicating that putative carriers for the neurological disorder have no cognitive deficit, it is likely the IQ alteration in siblings E II-8 and E II-9 is attributable to 3βHSD deficiency.

Learning difficulties have already been described in patients with 21 OHD CAH patients, probably due to hypoglycemia at presentation (47). We have noticed the same effects on intelligence in children with 21OHD CAH in our patients, with more than 20% of the children having a low IQ (44). This is probably due to the late presentation of our patients, who initially presented with severe hyponatremia and hypoglycemia. The intellectual deficit seen with 3βHSD2 deficiency in this series serves only to strengthen the case for setting up a national screening program for CAH in our country.

## Conclusions

3βHSD2 deficiency appears more prevalent in Algeria than elsewhere, with p.Pro222Gln the most frequent mutation. Mortality is high, with significant morbidity from PCOS and adrenal tumors in adolescence. IQ is usually in the lower half of the population range, with an increased risk of learning disability.

The diagnosis should be considered in all under-masculinized males with SW and healthy female patients with SW. Access to fludrocortisone is an important issue in our country and needs to be redressed urgently. The finding of adrenal masses in older patients with 3βHSD2 deficiency suggests adrenal hyperplasia requiring improved disease control rather than surgical intervention.

## Data Availability Statement

The raw data supporting the conclusions of this article will be made available by the authors, without undue reservation, on reasonable request.

## Ethics Statement

The studies involving human participants were reviewed and approved by the CHU Bab El Oued Ethical comittee. Written informed consent to participate in this study was provided by the participants’ legal guardian/next of kin. Written informed consent was obtained from the minor(s)’ legal guardian/next of kin for the publication of any potentially identifiable images or data included in this article.

## Author Contributions

AL designed and oversaw the study and wrote the manuscript. MD helped design and structure the manuscript and wrote the paper with AL. IP carried out the LC-MS/MS biochemistry studies and hormonal analyses in Lyon. ND performed the histological analysis and provided the pathology photographs. KM and KB examined the children and collected the data during the visits in 2021. VT oversaw the genetic analyses. DM carried out the genetic analyses. ZB oversaw the visits in 2021. YM carried out the genetic analyses and the LC-MS/MS biochemistry studies. FR-B supervised the hormonal analyses in Lyon, coordinated the genetic studies and helped write the manuscript with AL and MD. All authors listed have made a substantial, direct, and intellectual contribution to the work and approved it for publication.

## Funding

FR-B is supported by the Endocrinology Research Grant RECORDATI Rare Diseases/French Society of Endocrinology (SFE) 2020 and the Young Researchers 2021 grant of Lyon University Hospital (HCL).

## Conflict of Interest

The authors declare that the research was conducted in the absence of any commercial or financial relationships that could be construed as a potential conflict of interest.

## Publisher’s Note

All claims expressed in this article are solely those of the authors and do not necessarily represent those of their affiliated organizations, or those of the publisher, the editors and the reviewers. Any product that may be evaluated in this article, or claim that may be made by its manufacturer, is not guaranteed or endorsed by the publisher.
