# Supplementary material for: Genotype, Mortality, Morbidity, and Outcomes of 3β-Hydroxysteroid Dehydrogenase Deficiency in Algeria
Source: Front Endocrinol (Lausanne). 2022 Jun 10;13:867073. doi: 10.3389/fendo.2022.867073 (PMC9229600; doi:10.3389/fendo.2022.867073)
Supplement: Supplementary file 1 [file Table_1.docx]

Supplementary Table S1. Normative data for plasma steroids 17-hydoxyprogesterone (17-OHP), 17-hydroxypregnenolone, Δ-4 androstenedione, dehydroepiandrosterone (DHEA) DHEA sulphate (DHEA-S). Except for DHEA data from Kushnir et al, 2010 (reference 25) values derived from Lyon University Hospital, Bron-Lyon, France

| **Hormone** | **Age range** | **Reference range** |
| --- | --- | --- |
| **17OH-P**  (nmol/L) | Day 1 (both sexes)  Infants 1-3 months  - Male  - Female  Prepubertal age (both sexes)  Women (follicular phase women):  Men | 13.20 ± 5.90  6.08 ± 2.39  3.11 ± 1.48  1.18+/-0.69  <4.5  1.8 – 6.3 |
| **17-OH pregnenolone**  **(nmol/L)** | First year  All ages after First year | -  Basal: 0.13 to 13.7  After ACTH test: 0.42 to 46.6  NB Values > 90nmol/ml are indicative of 3‐β-hydroxysteroid dehydrogenase deficiency type II |
| **Δ4-androstenedione (nmol/L)** | 1 - 7 years  8 -11 years  Women  Men | 0.40 ± 0.22  1.30 ± 0.66  1.57 - 8.03  2.09- 5.93 |
| **DHEA (nmol/L)**  **Kushnir et al, 2010** | 6–24 months  Girls/Boys  2-7 years   7–9 years   10–11 years 1.5 - 13.2   12–13 years .1 - 21.7   14–15 years 4.3 - 24.5   16–17 years 5 - 31.4    Women 18–40 years/ >40 years  Men 18-40/40-67 years | 0.2-8.7  0.5-6.3/0.2-3.3  0.5 - 8.2/ 0.3-7.3  1.5-13.2/1.1-21.1  3.1/21.7/2 – 14.3  4.3 – 24.5/3.2-21.1  5-31.4/4.1-22.9  4.6 - 27.2 /2.2-16.4  4.6 – 27.2/2.2 – 16.4 |
| **DHEA – S (nmol/L)** | <1 week  1 to 4 weeks  1 to 12 months  1-4 years  5-9 years  Girls 10-14 years  Boys 10-14 years 660-6700 | 2930-16500  860-11700  90-3350  10-530  80-2310  920-7600  660-6700 |
